# Malignant Melanoma

**DOI:** 10.5402/2012/308279

**Published:** 2012-12-04

**Authors:** Aída Ortega Candil, Cristina Rodríguez Rey, José Luis Carreras Delgado

**Affiliations:** Department of Nuclear Medicine, Clinico San Carlos Hospital, 28040 Madrid, Spain

## Abstract

Nuclear medicine plays an essential role in the correct staging of patients suffering from melanoma. Both sentinel lymph node biopsy (SLNB) and positron emission tomography (PET) represent its main diagnostic tools. SLNB is the choice procedure for lymphatic regional staging of these patients, including the result of this technique in the 2002 American Joint Cancer Committee melanoma staging. SLNB sensitivity is superior than PET/CT for the detection of lymphatic micrometastases in early stages of the disease. PET/CT is mainly used in confirming clinical metastases suspected, detection of recurrences, and recurrence restaging. PET/CT has also shown superiority against conventional diagnostic methods in the detection of distant metastases, being able to detect illness even six months earlier than those methods.

## 1. Introduction

Cutaneous malignant melanoma (CMM) is the fifth most common cancer in men and the seventh in women and is one of the most deadly cancers [1]. In recent years there has been an exponential growth of melanoma cases. This increased incidence has created a medical alarm and undertakes to apply a multidisciplinary approach aimed primarily at prevention. Thus, despite the increased number of cases, mortality remains stable, probably related to improved diagnosis and early surgical treatment [2]. 

Suspicious lesions are characterized by asymmetry, border irregularities, colour heterogeneity, dynamics (dynamics in colours, elevation, or size), and “ABCD rule.” Dermatoscopy by an experienced physician enhances the diagnostic accuracy [3]. 

The prognostic factors of CMM include the thickness or Breslow index, Clark level, presence of ulceration, mitotic index, tumour location (located in the trunk and upper extremities have worse prognosis), the pattern of growth, histological type, age and sex, and involvement of regional lymph nodes. Of all of these parameters, the state of the first node draining from the tumour (called “sentinel node”) represents the most important prognostic factor in patients with melanoma in early stages [4].

Nuclear medicine plays a key role in the correct staging of CMM. Sentinel lymph node biopsy (SLNB) and positron emission tomography/computed tomography (PET/CT) represent its main diagnostic tools.

## 2. Sentinel Lymph Node Biopsy 

As previously discussed, the most important prognostic factor in patients with early-stage melanoma is the status of regional lymph nodes [5]. Because only approximately 20% of patients with an intermediate-thickness primary are expected to have metastases in the regional nodes, 80% of patients undergoing elective lymph node dissection (ELND) are at the risk for acute wound problems and the chronic morbidities of lymphedema, nerve injury, and anesthetic complications without actual survival benefit. SLNB was designed to identify occult nodal metastases and stage the regional nodal status. Initial experience with the SLNB was introduced in 1991 and published in 1992 by Morton et al. [6].

Lymphatic mapping relies on the hypothesis that the dermal lymphatic drainage from cutaneous sites to the regional lymph node basin is an orderly process and these lymphatic drainage patterns should mimic the metastatic spread of melanoma cells in the lymphatics. Therefore, the first lymph node(s) receiving lymphatic drainage (“the sentinel nodes”) are the most likely to contain metastatic disease [7]. A sentinel node (SN) is defined as “a node upon which lymph vessel originating in the tumour drains directly” [8]. 

SLNB is a multidisciplinary procedure that requires close cooperation between nuclear medicine physicians, surgeons, and pathologists for accuracy lymphatic mapping, dissection, and histopathologic analysis of the tumour draining nodes [9]. 

### 2.1. Staging of CMM

In 2001, the American Joint Committee on Cancer (AJCC) released a revised version of the staging system for CM (Tables 1 and 2), including the SLNB as part of staging. The AJCC staging system highlights the difference between clinical and pathologic staging. Therefore, clinical stage I and stage II disease refers to those patients with no evidence of nodal or distant metastases based on clinical and radiological evaluation. In contrast, pathologic staging is determined by information from both microstaging of the primary tumour and pathologic determination of lymph node (LN) status after either partial or complete lymphadenectomy [10]. 

In 2009, the AJCC made a new revision in the staging of melanoma that included a third category, the rate of mitosis, which was added to the existing tumour thickness and ulceration. The mitotic rate is important to distinguish between T1a and T1b tumours (when the mitotic rate is ≥1/mm^2^ is already a T1b tumour). Also in this new classification the presence of a single tumour cell in the SLNB is enough to classify as a stage III [11].

### 2.2. Clinical Indications

SLNB should be offered to patients with clinically localized disease and invasive melanoma, depending on different histopathologic characteristics of primary melanoma, including the following.

- Thickness ≤1 mm: In patients with melanomas of 0.76 to 1 mm thick SLNB indication differs according to different authors. Some authors systematically recommend SLNB because the risk of nodal metastasis is approximately 5%; others reserved it for cases with ulceration in the primary lesion in young patients or high mitotic rate [1]. In patients with a melanoma of ≤0.75 mm generally SLNB is not recommended since the risk of nodal metastases is around 1%.
- Intermediate thickness 1–4 mm: the risk of nodal metastasis increases from 8 to 30%, and there is a consensus that SLNB should be offered to these patients [12].
- Thickness >4 mm: these patients have the risk of distant metastases, and the risk of lymph node metastasis is around 40%; still the metastatic lymph nodes are usually not clinically palpable. Therefore, these patients also benefit from SLNB [13].

SLNB is also indicated when thickness of the primary tumour can not be determined with certainty.

Contraindications to SLNB include histologically confirmed metastatic LN and prior wide local excision [9].

### 2.3. Preoperative Lymphoscintigraphy

A variety of colloidal and soluble tracers have been used over the years for lymph studies and the radiopharmaceutical still varies among geographical regions. The main tracers are ^99m^Tc-human serum albumin (HAS) colloid in Europe, ^99m^Tc-sulphide colloide in North America, and ^99m^Tc-antimony trisulphide colloid in Australia. Usually the radiopharmaceutical is injected intradermally around the primary lesion or the scar from biopsy (4 injections) [8].

**Figure 1:**
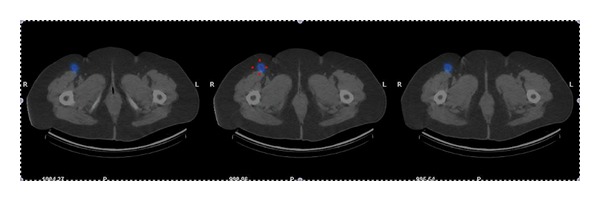
73-year-old woman with melanoma in the right leg (Breslow 3.8 mm) diagnosed in January 2011. It was resected in March of that year. PET/CT was performed in April 2011 with negative results. SLNB was undertaken in May 2011 (SPECT-CT images in axial view) showing a SN in right groin. Excision was performed with SN pathology negative for malignancy.

After the injection, dynamic (early) imaging and static (delay) imaging are recommended. In some centers sentinel lymphoscintigraphy is supplemented with SPECT/CT (single photon emission tomography/computer tomography), improving anatomical information [14, 15] (Figure 1). Vermeeren et al. indicate that SPECT/CT may detect additional LN in 16% of patients and that those nodes may be the only positive nodes [16].

The surface location of the SN should be marked on the skin. 

### 2.4. Intraoperative Lymphatic Mapping

Using the lymphoscintigram and the marked skin site as a road map, the surgeon moves a hand-held gamma probe over the regional nodal basin to confirm elevate radioactivity (a “hot spot”). After the incision is made over this hot spot, the hand-held gamma probe guides the surgeon in identifying radioactive node(s). Recently some centers are using portable gammacameras: Dengel et al. conducted a study of 20 patients with melanoma and found that the portable gammacamera was clinically useful in 20% of patients [17].

Many centers also inject prior to the surgery blue dye for better localization of SN. The blue dye may assist in visual conformation of the afferent lymphatics from the primary tumour site to the SN [8]. 

All SNs are excised and evaluated for metastases by permanent sectioning and staging with H & E and with IHC (antibodies to MART-1, HMB-45, and S-100) [11]. 

## 3. PET Technique

PET has become an essential imaging modality in the management of cancer patients. The radiotracer most frequently used is ^18^F-FDG (18-fluor-fluorodeoxyglucose). It is based on the ability to detect metabolic abnormalities associated to neoplastic processes that usually precede morphological changes. Nowadays PET/CT equipment combines metabolic and anatomic data in a unique procedure providing much more information. PET principles are based in the fact that malignant tumours with a high metabolic rate use as substrate glucose (and therefore FDG) in a higher proportion than surrounding tissues. PET studies are interpreted both qualitative and quantitative (using SUVmax parameter: Standardized Uptake Value). In the last decade PET/CT using ^18^F-FDG has become an essential tool in detection of malignant tumours and it has the advantage of assessing a whole body study in a single scan, an important fact in melanoma disease due to its high capacity to metastasize.

### 3.1. Indications

The potential indications of FDG-PET imaging in patients with melanoma are [18] the following.

1. Detection of regional and distant metastases at the time of initial diagnosis.
2. Surveillance in patients with a high risk for distant metastases based on extent of locoregional disease.
3. Patients with findings that are suspect for distant metastases.
4. Individuals with known distant tumour disease who still stand to benefit from customized therapies if new lesions are discovered or treated lesions regress.
5. Patients at high risk for systemic relapse who are considering aggressive medical therapy.
6. Evaluation of response to chemotherapy or immunotherapy.

### 3.2. PET/CT in Early Stages (Stage I and II AJCC)

In early stage melanoma PET/CT is not a useful tool as it has a low sensitivity in detecting microscopic lymphatic disease. SLNB becomes the election procedure for lymphatic regional staging in these patients. Its must also be considered that in these stages the risk of metastasic diseases is very low [19].

In regional melanoma staging, multiple studies have shown SLNB superiority against PET in T1 tumours [20, 21]. Jiménez-Requena et al. [22] underwent a meta-analysis concluding that PET is not useful in the evaluation of regional metastases as it cannot detect microscopic disease. PET spatial resolution for detection of tumoral tissue volumes fluctuates around 80 mm^3^, not being able to detect micrometastases [23]. Clark et al. [24] developed a prospective study recruiting 64 patients (T2–T4) and they suggested limited utility of PET in detection of occult melanoma metastases in initial staging. It would be a useful tool in evaluation of those patients presenting signs or symptoms of metastatic involvement, not being necessary in those asymptomatic undertaking SLNB. 

There are few studies defending PET in initial staging in patients with clinically localized melanoma. PET utility depends on the prevalence of detectable disease in the study population. Thereby PET diagnostic and clinical utility is superior in patients with advanced stages than in those with low prevalence of metastatic disease. Wagner et al. [25] undertook a prospective study in order to evaluate sensitivity and specificity of the technique in early stages population for detection of occult lymphatic metastases. They conclude that PET cannot replace SLNB in lymphatic staging. 

However Belhocine et al. [4] approve undertaking a PET scan in these early stages under certain clinical conditions, in combination of SLNB, as in the case of high risk melanomas (trunk and upper extremities location, Breslow > 4 mm, presence of ulceration and high mitotic index).

### 3.3. PET/CT in Advanced Stages (Stage III and IV AJCC)

Patients affected of melanoma stages III and IV of the AJCC, including those with resected metastases, have high risk of recurrent disease. Therefore, correct staging of this population is essential in order to determinate utility of potentially curative surgery or radiotherapy treatment.

**Figure 2:**
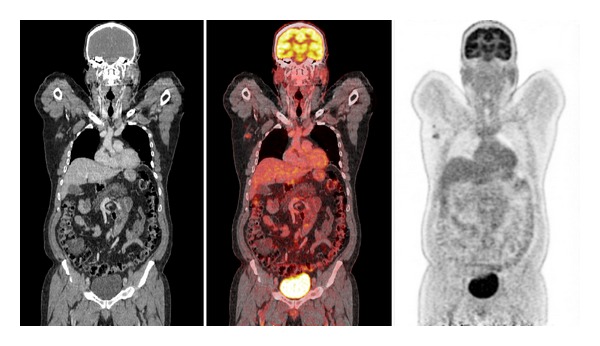
59-year-old man with right scapular melanoma (Breslow 2.4 mm) undertaking PET/CT scan for initial staging. Coronal view with pathologic right axillary nodes of 1.4 cm and SUVmax 7.7.

**Figure 3:**
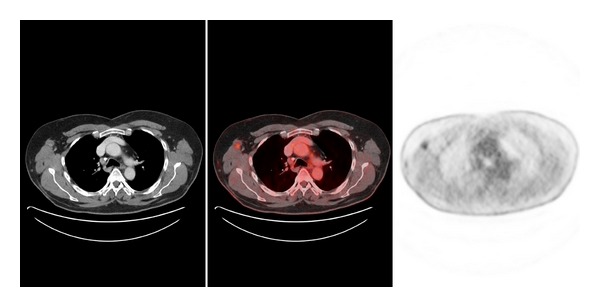
Axial view of the patient of Figure 2 showing the axillary nodes and subcutaneous implants.

**Figure 4:**
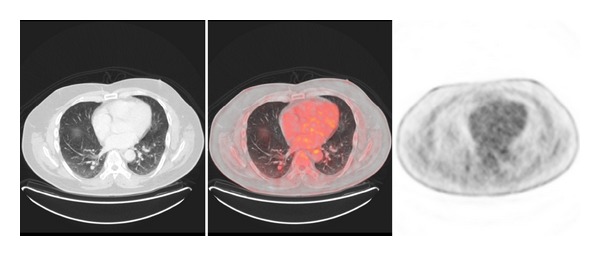
Axial view with pulmonary node in right lower lobe of 1.1 cm and SUVmax 2.5 suggestive of metastasis.

PET/CT has been shown to be more accurate than conventional modalities (CM) such as CT, magnetic resonance imaging (MRI), ultrasound, and physical examination for determining the existence and extension of metastatic disease. There are many studies that compared sensitivity and specificity of PET to CM in evaluating regional and distant metastases. The sensitivity of PET for metastatic lesions or abnormal regions ranged from 78% to 100%. PET/CT is able to detect disease even 6 months earlier than CM. Several studies that compare PET and CM in recurrent melanoma revealed a higher accuracy of PET in the detection of both local and distant metastases (Figures 2, 3, and 4) [26, 27]. Lesions missed in PET were due to its small size (less than 1 cm) or located in lung, liver, or brain. These false negative PET results can be detected with MRI; however with the new multimodality equipment (PET/CT) lung lesions are now easily identified.

We can conclude that PET/CT plays a key role in recurrent melanoma staging in combination with brain MRI for detection of brain metastases. PET/CT can also detect incidentally a second tumour, as it includes a whole body scan, having therefore an impact on patient management. Different series have reported changes in staging after PET scan ranging 12% and 34%. Impact in patient management from PET results (8%–61%) includes abandonment of planned surgical procedures, alteration of planned surgery either in extent or intent, and modifications in systemic therapy. From these results we can conclude that PET/CT should be performed in all patients suffering from melanoma before resection of regional or metastatic disease [10].

## 4. Conclusions

- Nuclear medicine plays an essential role in the correct staging of patients suffering from melanoma.
- SNLB is the technique of choice for detection of lymphatic metastases in early stages (Stage I and II AJCC), with higher sensitivity than PET/CT in the detection of lymphatic micrometastases.
- PET represents an imaging modality both sensitive and specific in detection of melanoma distant metastases and recurrent melanoma, especially in those patients undertaking surgery with curative intention.
- PET is more sensitive than CT in the detection of metastases located in subcutaneous tissue, lymphatic nodes, abdomen and bone; however PET could miss pulmonary metastatic disease, although this limitation is resolved with new multimodality equipment (PET/CT). Enhanced MRI represents the primary imaging modality for diagnosis of melanoma brain metastases.

## Figures and Tables

**Table 1:** TNM staging.

Primary tumour staging (T)	
Tx	Primary tumour is not identify
Tis	*In * *situ* melanoma
T1	≤1.00 mm
T1a	Without ulceration, without mitosis
T1b	With ulceration or mitotic rate ≥ 1 mm^2^
T2	1.01–2.00 mm
T2a	Without ulceration
T2b	With ulceration
T3	2.01–4.00 mm
T3a	Without ulceration
T3b	With ulceration
T4	>4.00 mm
T4a	Without ulceration
T4b	With ulceration

Lymph node status (N)	

N1	One node involved
N1a	Micrometastases
N1b	Macrometastases
N2	Two or Three nodes affected
N2a	Micrometastases
N2b	Macrometastases
N2c	Intransit metastases/satellites without metastatic nodes
N3	Four or more nodes or matted nodes or intransist metastases/satellites with metastastic nodes

Distant metastases (M)	

M1a	Distant skin, subcutaneous or nodal metastases with normal LHD levels
M1b	Lung metastases with normal LDH levels
M1c	All over visceral metastases or any distant metastases with elevated LDH levels

**Table 2:** 2002 AJCC Staging system for CM.

Stage AJCC	Clinical staging			Pathologic staging		
0	Tis	N0	M0	pTis	N0	M0

IA	T1a	N0	M0	pT1a	N0	M0
IB	T1b	N0	M0	pT1b	N0	M0
	T2a	N0	M0	pT2a	N0	M0

IIA	T2b	N0	M0	pT2b	N0	M0
	T3a	N0	M0	pT3a	N0	M0
						
IIB	T3b	N0	M0	pT3b	N0	M0
	T4a	N0	M0	pT4a	N0	M0
						
IIC	T4b	N0	M0	pT4b	N0	M0

IIIA	Any T	N1-3	M0	pT1-4a	N1a	M0
				pT1-4a	N2a	M0
						
				pT1-4b	N1a	M0
				pT1-4b	N2a	M0
IIIB	Any T	N1-3	M0	pT1-4a	N1b	M0
				pT1-4a	N2b	M0
				pT1-4a/b	N2c	M0
				pT1-4b	N1b	M0
						
IIIC	Any T	N1-3	M0	pT1-4b	N2b	M0

IV	Any T	Any N	M1	Any T	Any N	M1
